# Hereditary sensory and autonomic neuropathies: types II, III, and IV

**DOI:** 10.1186/1750-1172-2-39

**Published:** 2007-10-03

**Authors:** Felicia B Axelrod, Gabrielle Gold-von Simson

**Affiliations:** 1 Department of Pediatrics, New York University School of Medicine, New York, USA; 2 Department of Neurology, New York University School of Medicine, New York, USA

## Abstract

The hereditary sensory and autonomic neuropathies (HSAN) encompass a number of inherited disorders that are associated with sensory dysfunction (depressed reflexes, altered pain and temperature perception) and varying degrees of autonomic dysfunction (gastroesophageal reflux, postural hypotention, excessive sweating). Subsequent to the numerical classification of four distinct forms of HSAN that was proposed by Dyck and Ohta, additional entities continue to be described, so that identification and classification are ongoing. As a group, the HSAN are rare diseases that affect both sexes. HSAN III is almost exclusive to individuals of Eastern European Jewish extraction, with incidence of 1 per 3600 live births. Several hundred cases with HSAN IV have been reported. The worldwide prevalence of HSAN type II is very low. This review focuses on the description of three of the disorders, HSAN II through IV, that are characterized by autosomal recessive inheritance and onset at birth. These three forms of HSAN have been the most intensively studied, especially familial dysautonomia (Riley-Day syndrome or HSAN III), which is often used as a prototype for comparison to the other HSAN. Each HSAN disorder is likely caused by different genetic errors that affect specific aspects of small fiber neurodevelopment, which result in variable phenotypic expression. As genetic tests are routinely used for diagnostic confirmation of HSAN III only, other means of differentiating between the disorders is necessary. Diagnosis is based on the clinical features, the degree of both sensory and autonomic dysfunction, and biochemical evaluations, with pathologic examinations serving to further confirm differences. Treatments for all these disorders are supportive.

## Background

Although genetic disorders affecting the sensory and autonomic nervous systems are rare, their existence provides a means of furthering our knowledge regarding a very complex part of the nervous system. The close relationship between development and survival of the sensory and autonomic nervous system is especially well illustrated in the diversity of a group of genetic disorders known as Hereditary Sensory and Autonomic Neuropathies (HSAN) [1].

Classification of the various sensory disorders is unsettled and ongoing. In addition to the four numerical subtypes proposed by Dyck and Ohta [2], additional entities have been described [1,3] and there is semantic controversy. For example, familial dysautonomia (FD), originally the Riley Day syndrome [4], is also referred to as HSAN III and hereditary sensory neuropathy with anhidrosis is also referred to congenital insensitivity to pain with anhidrosis (CIPA) or HSAN IV (Table 1). Each HSAN disorder is likely caused by different genetic errors that affect specific aspects of small fiber neurodevelopment, which result in variable phenotypic expression. Because genetic tests are not available for all of the HSAN disorders, other means of differentiating between the disorders is necessary. Although specific neuropathological features have been described for some of the disorders, obtainment of neurological tissue is invasive and not always feasible. Thus clinical examinations with careful assessment of sensory and autonomic functions are preferable.

**Table 1:** Genetics of Hereditary Sensory and Autonomic Neuropathies (HSAN)

**Nomenclature**	* HSAN type	**Transmission**	**Chromosomal location**	**Gene**
Hereditary sensory radicular neuropathy	I	AD	9q22.1-22.3	*SPTLC1*
Congenital sensory neuropathy (CSN)	II	AR	12p13.33	*HSN2 (?)*
Familial dysautonomia (FD)/Riley Day	III	AR	9q31	*IKBKAP*
Congenital insensitivity to pain with anhidrosis (CIPA)	IV	AR	1q21-22	*NTRK1 *(TRKA)
Congenital insensitivity to pain with partial anhidrosis	V	NK	NK	*NTRK1*
Congenital autonomic dysfunction with universal pain loss (CAD)		NK	NK	
Progressive panneuropathy		NK	NK	

• * HSAN nomenclature from Dyck and Ohta [2]

• NK: not known, AR: autosomal recessive, AD: autosomal dominant

The genes have been cloned for HSAN types III and IV and autosomal recessive inheritance demonstrated. For type HSAN type II, new information is available suggesting that this disorder has a similar autosomal recessive inheritance. Penetrance is always complete in all three types but disease expression is variable. The impediment to neuronal development starts *in utero*, and thus clinical features such as poor suck, hypotonia, and temperature instability are usually present in the neonatal period. Furthermore, the lack of an axon flare in response to intradermal histamine is so universal and consistent in patients with HSAN II, III, and IV that it is considered one of the clinical diagnostic criteria. Nonetheless, diagnosis may be delayed in some individuals to late infancy or early childhood when injuries, enteric dysfunction, or delayed milestones become evident. Although the development of the unmyelinated neuronal population is impeded in all three HSAN types, the degree of involvement will vary with the particular HSAN type. Accordingly, pain and temperature perception and autonomic dysfunction will vary and also affect age of diagnosis and presentation (Table 2).

**Table 2:** Major clinical features of HSAN II, HSAN III, and HSAN IV

**Clinical features**	**HSAN type **II	**HSAN type **III	**HSAN type **IV
Onset	Birth	Birth	Birth
Initial symptoms (From birth to age 3 years)	Swallowing problems	Swallowing problems	Fevers
	Self mutilation (65%)	Aspiration pneumonia	Self mutilation (88%)
	Delayed development	Breech presentation (37%)	
		Hypothermia	
		Delayed development	
Unique features	No axon flare	No axon flare	No axon flare
	Lack of fungiform papilla	Lack of fungiform papilla	Anhydrosis
	Hearing loss (30%)	Alacrima	Consanguinity 50%
**Sensory dysfunction**			
Depressed DTR	Frequent (71%)	Almost consistent (99%)	Infrequent (9%)
Pain perception	Absent	Mild to moderate decrease	Absent
Temperature perception	Severe decrease	Mild to moderate decrease	Absent
Vibration sense	Normal	Normal	Normal to moderate decrease
**Autonomic**			
Gastroesophageal Reflux	Frequent (71%)	Frequent (67%)	Uncommon (24%)
Postural hypotension	Uncommon (25%)	Almost consistent (99%)	Uncommon (29%)
Episodic hypertension	Rare	Frequent	Rare
**Ectodermal features**			
Dry Skin	No	No	Consistent
Fractures	29%	40%	71%
Scoliosis	59%	85%	23%
**Intelligence**			
IQ < 65	Common (38%)	Uncommon (10%)	Common (33%)
Hyperactivity	Common (41%)	Uncommon	Common (54%)

Rare < 1%

Infrequent < 10%

Uncommon < 30%

Common 30–65%

Frequent > 65%

DTR = deep tendon reflexes

This review discusses diagnosis, neuropathology, and clinical expression of the HSAN disorders in general, as well as the specific characteristics that help differentiate the three most common disorders, HSAN II through IV, as they have been the most intensively studied.

## Genetics

With the exception of hereditary sensory radicular neuropathy (HSAN I), which presents in the second decade and is transmitted as a dominant disorder, the other HSAN are present at birth and are transmitted as autosomal recessive disorders (Table 1). HSAN I has been associated with a mutation in the *SPTLC1 *gene which encodes for serine palmitoyltransferase – the rate limiting enzyme for the synthesis of the sphingolipids, ceramide and sphingomyelin. [5,6]. Other HSAN with specific genetic mutations are HSAN III (FD) and HSAN IV (CIPA), which are transmitted as autosomal recessive disorders.

FD is almost exclusive to individuals of Eastern European Jewish extraction [7-9]. In this population the carrier rate is estimated as approximately 1 in 30 with a disease incidence of 1 per 3600 live births [10]. In 1993 the FD gene was localized to the long arm of chromosome 9 (9q31) [11]. In 2001 the gene was cloned. A single point mutation was identified on the *IKBKAP *gene and greater than 99% of affected individuals are homozygous for this common mutation that occurs within intron 20. The result is tissue-specific mis-splicing which results in decreased amounts of normal transcript, especially in neuronal tissue. Therefore, there is a predicted lack of expression of the normal protein product, IKAP [7,8].

IKAP is a subunit of the highly conserved complex Elongator, which is involved in transcriptional elongation. Recently, RNA interference studies have shown that depletion of IKAP, and thus Elongator, results in reduced transcriptional elongation of several target genes via histone H3 hypoacetylation. A subset of these target genes are required for cell motility, and FD patient cells were shown to be defective in cell migration assays, suggesting that defective cellular motility may underlie the developmental neuropathology of FD. Furthermore, studies in yeast suggest that the Elongator complex is involved in exocytosis and tRNA modification and studies in mammalian cells implicate IKAP as a scaffold protein involved in cytoplasmic JNK activation in response to extracellular stress. IKAP's role in these processes, and how they relate to the FD phenotype, is not yet understood.

Two other missense mutations that cause FD have been reported. One mutation, in exon 19, was noted in four unrelated Jewish patients heterozygous for the major splice mutation [7,8]; the second mutation, in exon 26, was reported in a single patient who was also heterozygous for the major splice mutation but inherited the new mutation from a non-Jewish parent [9]. FD is the only HSAN for which genetic testing is commercially available.

HSAN IV is caused by mutations in the *NTRK1 *(TRKA) gene that is located on chromosome 1 (1q21-q22). This gene encodes for neurotrophic tyrosine kinase receptor type 1 that is autophosphorylated in response to NGF (nerve growth factor) [12]. As a result of loss of function mutations, signal transduction at the NGF receptor is impeded and NGF dependent neurons, the small sensory and sympathetic neurons, fail to survive. There does not seem to be a particular ethnic distribution for this recessive disorder but onehalf of reported cases have occurred in consanguineous marriages [1,12,13]. As many mutations have been described, commercial molecular genetic diagnostic testing is not yet feasible.

Specific genetic mutations have not been identified for the other HSAN disorders, although there is some evidence that a mutation in the *NTRK1 *gene also may be responsible for HSAN V [14].

The other HSANs including HSAN II, are presumed to be autosomal recessive disorders or *de novo *dominant mutations. In each of the disorders, other siblings but not parents have been affected. None of the disorders has a sex preference or particular ethnic distribution.

## Clinical features, diagnosis and treatment

**Figure 1:**
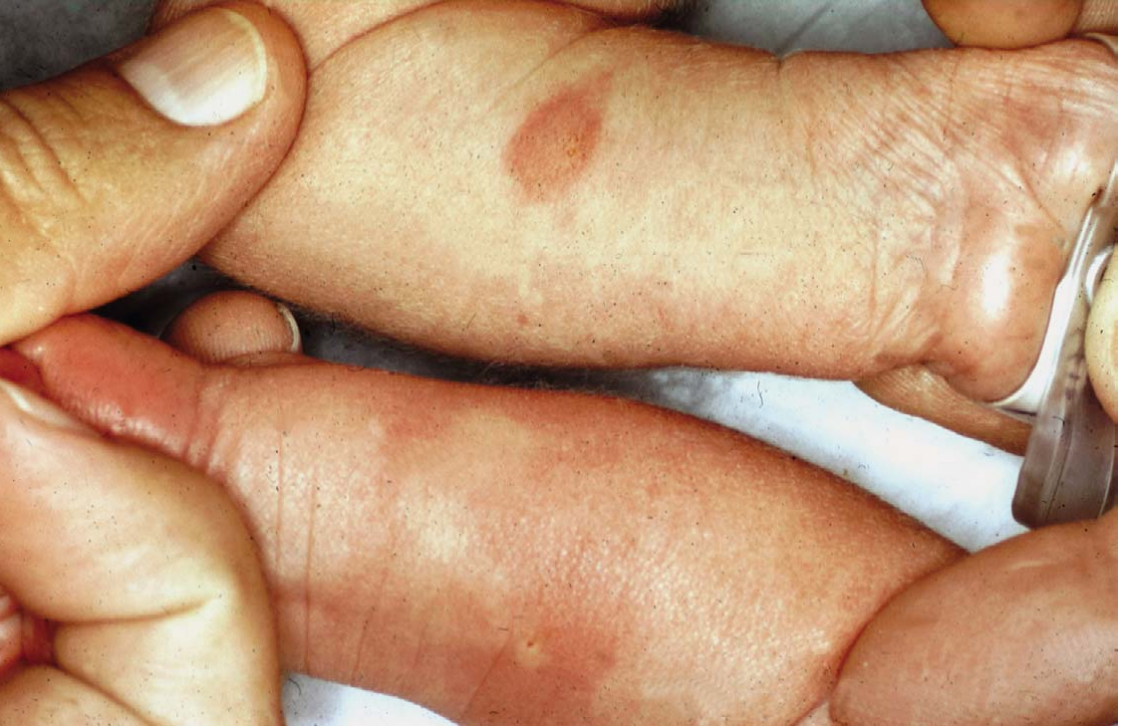
Histamine test. Dysautonomic reaction (forearm on top) demonstrates a narrow areola surrounding the wheal. Normal reaction (lower forearm) displays diffuse axon flare around a central wheal.

For each of the HSAN disorders, penetrance is complete but there can be marked variability in expression. Diagnosis is based upon clinical features as well as the degree of both sensory and autonomic dysfunction. In order to differentiate between the HSAN, quantitative assessment of function as well as identification of particular clinical characteristics is required. Unique to all HSAN, is lack of a normal axon flare in response to intradermal histamine phosphate (Figure 1). As can been seen in Figure 1, when a quantity of 0.1 ml of histamine is injected intradermally using a fine needle and tuberculin syringe (concentration = 0.275 mg histamine phosphate/ml) in a normal subject, the result is a bright red histamine flare due to capillary vasodilatation within five minutes. This normal effect is due to axon reflex within dermal nerves. Because the normal axon flare response depends on activation of unmyelinated C-fibers, in some mild cases of HSAN II patients who have largely preserved C-fibers or some HSAN V patients who have little reduction of C-fibers, the histamine test may be normal. However, there are other features that assist in distinguishing the disorders (Table 2).

## Congenital sensory neuropathy (HSAN II)

### Clinical presentation

HSAN II presents in infancy or early childhood and is nonprogressive. It is characterized by profound and universal sensory loss and marked hypotonia. HSAN II occurs sporadically or with autosomal recessive inheritance. There is no sex preference or particular ethnic preponderance, and to date there is no increased incidence of consanguinity.

Although the disorder is associated with profound sensory loss involving large and small fiber modalities, the earliest problems are caused by autonomic dysfunction [1-3,15-17]. The neonatal course is characterized by severe feeding problems and frequent apnea [15-17]. Gastroesophageal reflux occurs commonly. Other autonomic disturbances, such as erythematous blotching of the skin and postural hypotension, have not been observed. Episodic hyperhydrosis as well as patchy areas of anhidrosis can occur in the same patient. Overflow tearing is frequently delayed but is eventually normal. Pupils have an exaggerated response to parasympathomimetic agents.

All peripheral sensations are affected but distribution of somatic involvement may vary. Pain, temperature, and position senses are involved. Trophic changes are present in the upper and lower extremities. At one time, HSAN II was termed *'Morvan's disease' *(after the physician who first described the disease and the occurrence of *'painless whitlows' *or acrodystrophic neuropathy [18,19]. HSAN II is associated with frequent occurrence of unrecognized injuries and fractures of hands, feet, and limbs, as well as Charcot joints [1,17,19].

Selfmutilation may begin as early as four months of age and is usually associated with eruption of primary dentition [20]. Taste sensation is diminished and lingual fungiform papillae are hypotrophic. Corneal and gag reflexes may be diminished. Deep tendon reflexes are attenuated and hypotonia is common which delays attainment of developmental milestones and may contribute to scoliosis. Despite the marked sensory abnormalities, other aspects of the neurological examination may be normal including mental function, cranial nerves, cerebellar and motor functions. Although the tendon reflexes are decreased or absent, there is no muscle atrophy or muscle weakness.

### Pathology

Sural nerve biopsy reveals a marked reduction in nerve size and depletion of large and small myelinated fibers but only a slightly decreased number of unmyelinated fibers [16,19]. No cutaneous sensory receptors or nerve fibers are seen but catecholaminergic sympathetic fibers can be demonstrated by aldehydeinduced fluorescence [19].

### Diagnosis

As there is no DNA molecular testing available, diagnosis is based on documenting profound peripheral sensory involvement of both peripheral and cranial nerves, which includes an absent axon flare after intradermal histamine. Clinical identification is made by the finding of a mutilating acropathy with a severe, distally pronounced impairment of all sensory qualities that includes light touch sensation, position sense and vibratory perception, as well as pain and temperature perception. Supportive evidence includes selfmutilation, hypotonia and delayed milestones, and normal somatic growth.

The neurophysiological evaluation shows abnormal vibratory thresholds, and quantitative sensory testing may also reveal elevated thermal thresholds at the hands and feet [16]. Typically, nerve conduction studies confirm severe impairment of sensory nerve conduction velocities and absent sensory nerve action potentials, but motor nerve conduction velocities are at or slightly below the normal limit and compound motor action potentials may only have slightly reduced amplitudes [16,19]. According to Dyck, the electromyogram (EMG) shows minimal fibrillations and a rarified pattern with maximal volitional innervation as well as an increased number of polyphasic motor action unit potentials [16]. Electroencephalographic (EEG) studies are normal, but auditory evoked potentials indicate immature pathways.

### Management

Management is symptomatic and preventative. If feeding problems compromise nutrition and if gastroesophageal reflux is also present, fundoplication with gastrostomy is recommended. Sleep pneumograms can determine if there is central apnea and if respiratory support is needed. Parents and patients education is required to learn how to avoid injury and to be alert for signs of unrecognized trauma.

## Familial dysautonomia (FD, HSAN III, or Riley-Day syndrome)

### Clinical Presentation

**Figure 2:**
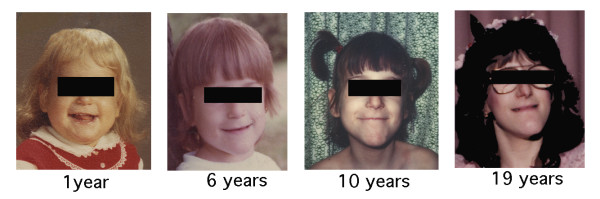
Facial features. Facial features of a patient with HSAN over time. Note flattening of upper lip. By age 10 years prominence of lower jaw is apparent and by age 19 years there is mild erosion of right nostril due to inadvertent self-mutilation.

**Figure 3:**
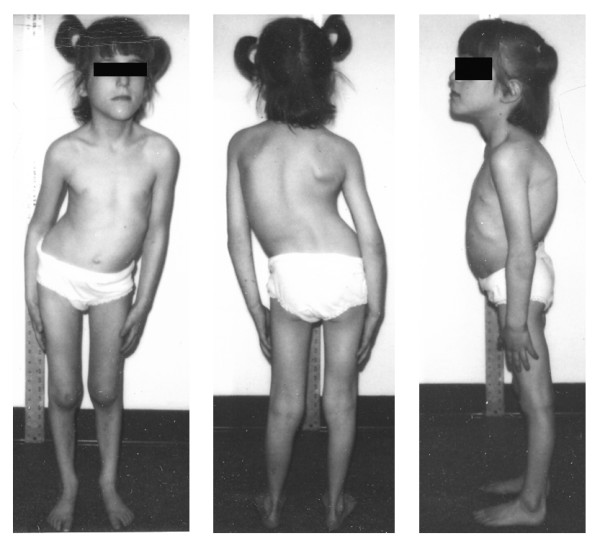
Kyphoscoliosis in a 10-year-old girl with HSAN III.

HSAN III is the most common of the HSAN disorders. It is present at birth and it is progressive [21,22]. Because the effects of the autonomic nervous system are pervasive, there are protean secondary functional abnormalities that result in multisysytem dysfunction. Clinical expression varies widely from individual to individual as does disease progression, even within the same family. The child with familial dysautonomia is not born with any obvious dysmorphism but a characteristic facial expression may develop over time that appears to be due to the effects of facial tone on bone development. The facies appear more similar with advancing age and there is a curious flattening of the upper lip that is most obvious when smiling (Figure 2). Other common physical characteristics are due to orthopedic problems such as severe kyphoscoliosis and short stature (Figure 3).

Autonomic manifestations are prominent, involve peripheral and central tracts, and impose the greatest impediments to function [22]. Although absence of tears (alacrima) with emotional crying is one of the cardinal features of the disorder, this sign may not be immediately recognized since lack of overflow tears is normal until about seven months of age. Thus the earliest sign of autonomic dysfunction usually is feeding difficulties. Oral incoordination results in a poor suck and misdirection of the bolus and gastroesophageal reflux cause recurrent aspiration pneumonia that can result eventually in chronic lung disease. Compounding the problem of aspiration pneumonias and chronic lung disease is the presence of relative insensitivity to hypoxemia [23-26], which limits ability to cope with pneumonia or even travel to high altitudes where there is reduced partial pressure of oxygen. Ensuing hypoxemia may lead to hypotension, bradyarrhythmia and even syncope. FD patients also exhibit protracted episodes of nausea and vomiting that are termed the '*dysautonomic crisis'*, a constellation of symptoms with abnormal gastrointestinal motility being only one aspect. During the crisis there are cardiovascular changes including hypertension, tachycardia and blotching. There is a generalized increase in secretions resulting in excessive diaphoresis, gastrorrhea, bronchorrhea and hypersalivation with copious drooling. In addition there are negative personality changes that can range from irritability and withdrawal to general excitation with pacing and picking at the skin. Forty percent of individuals with FD manifest a cyclical crisis pattern with frequencies that can occur daily, weekly, or monthly. In these individuals the crisis is most likely to follow morning arousal. However, with sufficient stress, a crisis can be observed in any affected individual. The stress can be emotional secondary to anxiety or the stress can be physiologic secondary to infection, surgery, sleep deprivation, excessive fatigue, or visceral pain secondary to constipation or menses.

The individual with familial dysautonomia commonly experiences orthostatic hypotension without a compensatory tachycardia, as well as episodic and supine hypertension [22,27]. Orthostatic change in blood pressure is a cardinal sign of autonomic insufficiency. In FD individuals, the lack of compensatory tachycardia is believed due to decreased sympathetic innervation of the vasculature with subsequent inability to increase plasma norepinephrine in response to standing or exercise [28]. Clinical manifestations of orthostatic hypotension include presyncope: episodes of lightheadedness, dizzy spells, and blurring of vision. Some patients complain of "weak legs." On occasion, there may be syncope. Symptoms tend to be worse in the morning, in hot or humid weather, when the bladder is full, before a large bowel movement, after a long car ride, coming out of a movie theater, or with fatigue. Infection and dehydration can compound symptoms. Generally, low blood pressure is more troublesome in the adult years and can greatly limit function and mobility.

The dysautonomic patient can also experience episodic hypertension in response to emotional stress or visceral pain or as part of the crisis constellation. The episodic hypertension is thought to be caused by central release of catecholamines and exaggerated peripheral responses due to denervation hypersensitivity, as supersensitivity to cholinergic and adrenergic agents has been demonstrated [29,30]. Hypertension is also more apt to occur when the patient is supine and again this may be due to exaggerated responses due to denervation hypersensitivity as the norepinephrine levels are not extraordinarily high in this position. Further manifestations of vasomotor and cardiovascular perturbations include erythematous skin blotching and hyperhydrosis with excitation or even eating.

The sensory abnormalities in individuals with FD are not as profound as with the other HSAN disorders and self-mutilation is rare. Pain and temperature perception are decreased but not absent [21,31]. While bone and skin pain are poorly perceived, sensitivity to visceral pain has been observed. There are appropriate responses to pleural effusions, esophageal irritation, and menstrual cramping. Corneal and tendon reflexes are hypoactive, and taste appreciation is diminished which is due to absence of lingual fungiform papillae. Later in the course of the illness, vibratory sensory loss and impaired coordination appear [21]. Somatic growth is poor and by 10 years of age, 85% of patients have scoliosis [32] (Figure 3).

Developmental milestones are commonly delayed as a result of infantile hypotonia and poorly graded movements. Although receptive language is usually normal, expressive speech is delayed and often is poorly articulated or nasal in quality. Performance on standardized intelligence tests frequently demonstrates better verbal than motor performance [33]. Executive planning and organizational skills tend to be poor. Seizures with decerebrate posturing can follow breathholding even in children with normal EEG findings.

There is pathological and clinical evidence that FD is associated with neurological progression. The question is whether this progression is inherent to the disorder or is secondary to some of the other problems such as cardiovascular lability. In older patients, central neuropathology has not been documented but Magnetic Resonance Imaging (MRI's) frequently show generalized atrophy including the cerebellum. This may contribute to worsening of the ataxic gait and greater balance problems requiring the use of walkers or wheelchairs in the older patients. In some FD adults changes have also been noted in mental processing that can range from poor concentration to phobias to the rare occurrence of actual dementia [34]. There is also progressive optic nerve atrophy that severely limits vision in the older patients [35].

It is possible that compromised cerebral perfusion may contribute to the neurological progression with age as other cardiovascular perturbations worsen with age such as more pronounced problems with postural hypotension and worsening supine hypertension, development of ischemic glomerulosclerosis [36] and cardiac arrhythmias [37]. Supporting this hypothesis are the observation by Hilz *et al *of decreased or inappropriate cerebral blood flow under various conditions including tilt as well as hypoxia and hypercapnia [26,38,39]. Thus some of the neurological progression might be avoided with better management of blood pressure lability.

Fertility in both males and females has been proven, and offspring of affected individuals have been normal [40]. Progressive diminution of renal function with age is a frequent observation [36,41]. Moderate azotemia is an early sign. Creatinine clearance decreases, and many patients have subnormal renin excretion. Nephrosclerosis has been reported on autopsy [36]. Despite these multiple problems, with improved supportive and preventative measures about half of the patients now reach adulthood [35,42].

### Pathology

Familial dysautonomia was originally reported as "central autonomic dysfunction with absent lacrimation" [4], yet consistent neuropathological findings all have been restricted to the periphery [43] and the information derived suggests that familial dysautonomia results from incomplete development of the sensory and autonomic systems, as well as limited survival of sensory and autonomic neurons, with sympathetic development more widely affected than parasympathetic. Overall there are decreased numbers of unmyelinated and small myelinated neurons. The sural nerve is hypoplastic and contains markedly reduced populations of nonmyelinated and small myelinated fibers, and there is lesser depletion of largemyelinated axons [44,45]. Neuron somas in the Gasserian and spinal ganglia are reduced to 50% of the normal number and neuron somas in cervical and thoracic sympathetic ganglia are reduced to 27%–37% [43,46]. The reduced size of cervical and thoracic ganglia is consistent with cardiac autonomic dysfunction. Sympathetic terminals are sparse or absent on vessels of skin, which probably contributes to vasomotor lability [47,48]. Increasing residual nodules and decreasing neurons in older patients indicate a degenerative component. The dorsal columns conducting vibration, proprioceptive and touch information are initially normal but diminish markedly in older patients [46]. Parasympathetic ganglia are variably affected; the ciliary ganglion is normal or marginally depleted of neurons but the sphenopalatine ganglion has less than one tenth of that in the normal population. Consistent qualitative central nervous system (CNS) anomalies have not been found. However, there is increasing clinical evidence for central autonomic dysfunction and new genetic findings indicate that central regions have altered expression of the normal gene product [49]. Thus we assume that pathologic confirmation of central defects will eventually be provided.

### Diagnosis

**Figure 4:**
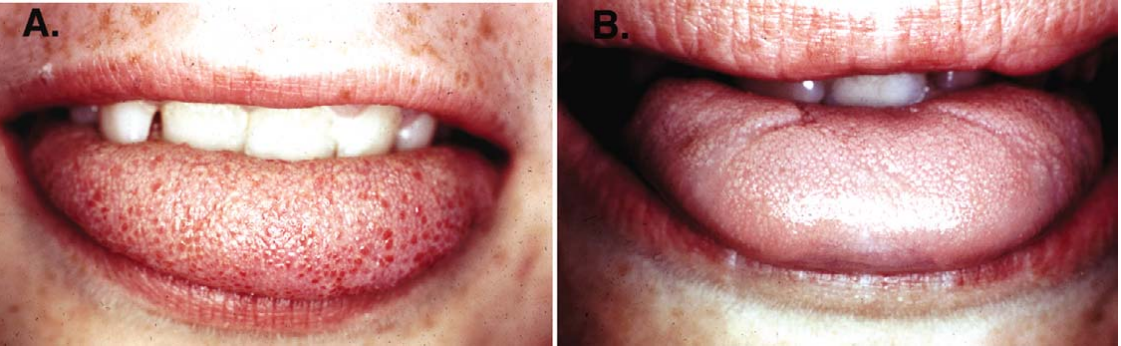
A. Normal tongue with fungiform papillae present on the tip. B. Dysautonomic tongue. Note the absence of the highly vascularized fungiform papillae from the tongue tip, which gives the appearance of a smooth tongue.

Diagnosis is based upon clinical recognition of both sensory and autonomic dysfunction. The combination of the "cardinal" criteria, *i.e. *alacrima, absent fungiform papillae (Figure 4), depressed patellar reflexes, and abnormal histamine test (Figure 1) in an individual of Ashkenazi Jewish extraction is usually sufficient to make the diagnosis.

As individuals affected with the other HSANs also fail to produce an axon flare after intradermal histamine [1], careful assessment of the other clinical signs and symptoms is necessary in order to distinguish between these disorders. Because there can be extreme variability in expression, clinical criteria are not always sufficient so that DNA molecular diagnostics should be performed to provide a definitive diagnosis. FD is the only HSAN for which commercially available genetic testing is available.

### Management

Although gene identification holds the promise of eventually yielding more specific treatments for this disorder, at present treatments remain supportive and directed toward specific problems that vary considerably from patient to patient [50,51]. In particular there are four areas that remain extremely problematic as they can severely limit independent function. These are alacrima, gastrointestinal dysfunction, respiratory dysfunction, and blood pressure lability. These problems continue to challenge the treating physician and provide the impetus to find definitive therapy for this disorder.

#### Alacrima

Alacrima in combination with corneal analgesia predispose the eye to development of corneal ulcerations and scarring. Frequent administration of topical lubricants is used to prevent corneal complications [35]. Cautery of the tear ducts also helps to increase baseline moisture. For the most part, corneal transplants have had a poor success rate.

#### Gastrointestinal dysfunction

Gastrointestinal dysmotility includes oropharyngeal incoordination, abnormal esophageal peristalsis, erratic gastric emptying, gastroesophageal reflux and the episodic protracted vomiting attacks termed "dysautonomic crises". Fundoplication with gastrostomy is often necessary to compensate for the oral incoordination and gastroesophageal reflux as the procedure helps maintain adequate caloric intake, avoids dehydration, and minimize aspiration. Treatment of the dysautonomic crisis is a greater challenge because therapeutic intervention is two-fold, directed toward the brain as well as the gastrointestinal tract. Many of the triggers are induced by central insults, but the prominent manifestations are gastrointestinal. Thus the concept has been proposed that the crisis may represent a type of autonomic seizure and that nausea may be the aura [52]. This hypothesis was supported by SPECT studies demonstrating areas of enhanced perfusion in the temporal area during crises. The hypothesis that crisis is a type of autonomic seizure is consistent with the clinical experience; typical antiemetic medications are not as effective in treating the episodes. However, centrally acting agents such as the benzodiazepines lock the GABA_A _receptor into a conformation where the neurotransmitter GABA has a much higher affinity for the receptor, thus increasing the frequency of opening of the associated Chloride ion channel and hyperpolarizing the membrane. This action potentiates the inhibitory effect of the available GABA leading to sedatory and anxiolytic effects. Thus the medications that are used to control the crisis are GABA enhancing agents central alpha-adrenergic agonists such as clonidine, and anticholinergics such as cyproheptadine [50,51].

#### Respiratory dysfunction

There are three potential factors that can compromise respiratory function and contribute to increased morbidity and mortality in the individual with familial dysautonomia – chronic lung disease secondary to repeated aspirations, restrictive lung disease imposed by scoliosis and muscle weakness, and chemoreceptor dysfunction resulting in blunted responses to hypoxemia.

The most effective means of avoiding chronic lung disease secondary to aspiration is to carefully assess oral coordination. Cineradiographic swallowing studies using various food consistencies often demonstrate that thin liquids are more apt to be aspirated than softer foods or thickened liquids. Cineradiographic studies, or esophageal pH studies, provide further information regarding gastroesophageal reflux, which is another potential source of aspiration pneumonia. Due to the frequent combination of oral incoordination and gastroesophageal reflux, medical management with prokinetic agents, proton pump inhibitors, thickening of feeds, and even positioning often is not adequate. Thus fundoplication with gastrostomy is commonly recommended [50,51].

Restrictive lung disease is imposed by the frequent occurrence of spinal curvature. Annual examination of the spine should be implemented to prevent severe deforming curves and exercise of the hypotonic intercostal muscles is essential for maintaining adequate vital capacity. If brace therapy is not successful in halting progression, or if the brace itself inhibits respiratory excursion, or if the curve is severe, spinal fusion is recommended [32,50].

Another component of respiratory dysfunction is decreased chemoreceptor and baroreceptor sensitivity, which severely compromises ability to cope with respiratory infections, and other potential causes of hypoxia [23-26]. FD individuals do not appropriately increase minute ventilation in hypoxic or hypercapnic situations such as high altitudes, pressurized airplane cabins or with intercurrent pneumonia. Even underwater swimming poses a potential threat. Therefore a high index of suspicion for pneumonia should be maintained rather than attributing febrile episodes to autonomic dysfunction with temperature dysregulation. Appropriate instructions need to be given regarding air travel and travel to high altitudes.

#### Blood pressure lability

The individual with familial dysautonomia commonly experiences postural hypotension without compensatory tachycardia as well as episodic hypertension and supine hypertension [27]. Treatments for orthostatic hypotension include hydration, exercise and use of various medications [50,51]. Adequate hydration can also be monitored by blood urea nitrogen levels. Lower extremity exercises are encouraged to increase muscle tone and promote venous return. Other treatments have included wearing of elastic stockings, additional salt in the diet, and use of fludrocortisone, a mineralocorticoid, and midodrine, a peripheral alpha adrenergic agonist [53,54]. Fludrocortisone 0.1 mg is given in the morning. A second dose is often added at midday. Midodrine is also given in the morning at 0.05–0.1 mg/kg per dose and is usually repeated every 4 hours. It should be stopped within four hours of bedtime.

Hypertension appears to be better tolerated than hypotension. As blood pressure is so labile and so susceptible to positional changes, treatment for hypertension should be instituted only when hypertension is refractory or associated with headache or symptoms of crisis, such as nausea or agitated activity. At such times, treatment is usually instituted with diazepam or clonidine [50,51].

## Congenital insensitivity to pain with anhidrosis (CIPA; HSAN IV)

### Clinical Presentation

HSAN IV is the second most common HSAN and like the other recessively transmitted HSAN onset is in infancy [1-3]. Several hundreds cases of HSAN IV have been published. What distinguishes HSAN IV is the extensive involvement of ectodermal structures including skin, bone and nervous system, especially absent or markedly decreased sweating, the cardinal feature of the disorder.

HSAN type IV is characterized by anhidrosis, absent or markedly decreased sweating [1-3,12,13,55-58]. It is the anhidrosis that causes episodic fevers and extreme hyperpyrexia that is usually the earliest sign of the disorder and can cause recurrent febrile convulsions secondary to high environmental temperature [1,12]. The anhidrosis is probably secondary to impaired thoracolumbar sympathetic outflow. It is present on the trunk and upper extremities in 100% of cases, whereas other areas of the body are variably affected. It causes the skin to become thick and callused with lichenification of palms, dystrophic nails, and areas of hypotrichosis on the scalp [12,13,56,57].

**Figure 5:**
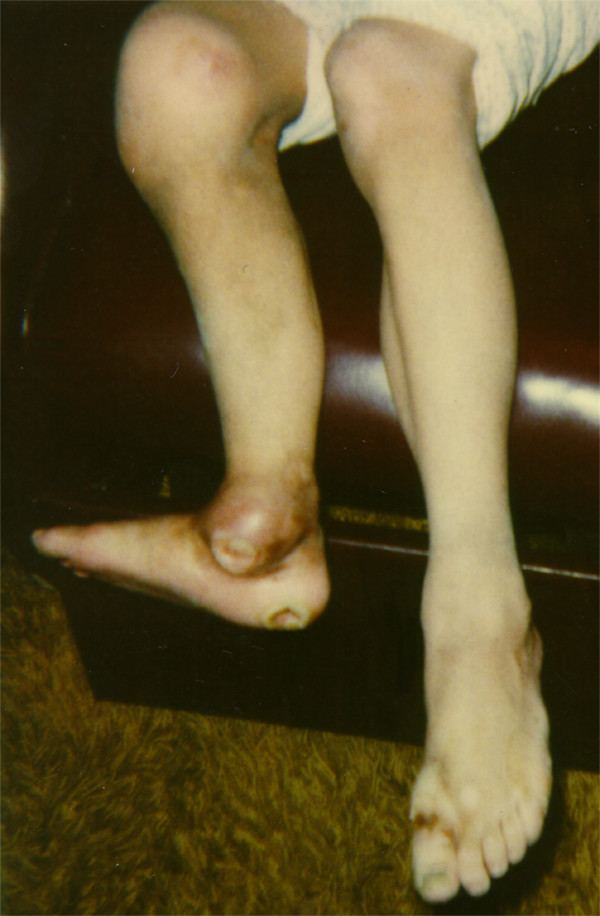
Charcot joints are demonstrated in this boy with HSAN IV or congenital insensitivity to pain with anhidrosis (CIPA). The left knee and left ankle are enlarged and distorted. The skin over the medial aspect of the ankle is darkened with a draining would secondary to superimposed osteomyelitis. There are other areas of trauma and ulcers including a site on the left heel.

Like FD, HSAN IV is associated with decreased sensation and autonomic dysfunction. It may be confused with FD in the neonatal period but the differences become much clearer with time as the characteristic anhidrosis causes cutaneous changes and the sensory insensitivity is much more profound resulting in self-mutilation, auto-amputation, and corneal scarring [1,3,12,56,57]. Although there is no immunological problem, individuals with HSAN IV have definite problems in healing of ectodermal structures – skin and bone. Fractures are slow to heal and large weight bearing joints appear particularly susceptible to repeated trauma and frequently go on to the development of Charcot joints and osteomyelitis (Figure 5). Speech is usually clear. Although hypotonia and delayed developmental milestones are frequent in the early years, strength and tone normalize with age. However, there can be severe learning problems. Hyperactivity and emotional lability are common [1,3,12,56].

Other than the characteristic anhidrosis, other autonomic perturbations are mild to absent. Although patients exhibit myosis with dilute intraocular mecholyl, emotional tearing is normal [1,3]. Postural hypotension with compensatory tachycardia may be present but not episodic hypertension suggesting that the blood pressure problems are secondary to disuse atrophy rather than sympathetic dysfunction. There is none to minimal gastrointestinal dysmotility; vomiting is not a feature of the disease and cyclical crises do not occur. Insensitivity to hypoxia and hypercapnia has not been noted. However, patients may have a heightened sympathetic response, as erythematous blotching of the skin with excitement has been seen in younger patients. Acrocyanosis is notably lacking. Gastrointestinal dysmotility is infrequent; vomiting is not a feature of the disease and cyclical crises do not occur [1,3]. Insensitivity to hypoxia and hypercapnia has not been noted.

### Pathology

Neuropathological studies have demonstrated decreased neuronal populations. Sural nerve biopsies show myelinated nerve fibers are present but unmyelinated fibers are absent [2]. Skin biopsy morphology of HSAN IV patients reveals deficient C and Aδ fibers in the epidermis and absent or hypoplastic dermal sweat glands without innervation [59-62].

Although sweat glands can be demonstrated in skin biopsies, unmyelinated fibers are not adjacent to sweat glands [2,59-62]. The lack of sweat gland innervation accounts for the clinical manifestation of severe anhidrosis and the objective absence of sympathetic skin responses (SSR) [63]. Although central neuropathology has not been demonstrated for HSAN type IV, affected individuals have clinical features suggesting central involvement as the patients are frequently exhibit hypotonia and delayed developmental milestones in the early years and there can be severe learning problems, often associated with hyperactivity [1,12,58].

### Diagnosis

It has been suggested that diagnosis of this disorder requires three clinical criteria, *i.e. *anhidrosis, decreased pain perception and mental retardation [12]. However, our experience indicates that the degree of expression of the three clinical features, especially intellectual ability, can be extremely varied. In addition to the absence of an axon flare following intradermal histamine, which is common to all the HSAN, clinical diagnosis is primarily based on the fact that HSAN IV is the only HSAN that is associated with widespread anhidrosis. The sensory abnormalities are also profound and widespread. They include cranial nerves and visceral sensation resulting in insensitivity to superficial as well as deep, visceral painful stimuli and in impressive self-mutilation and traumatized joints. Temperature sensation is also decreased or absent but deep tendon reflexes are usually intact. Although there has been gene identification, numerous mutations does not allow simple DNA diagnosis so that it is not routinely used for clinical confirmation of diagnosis [12,13]. Penetrance is complete, but expression varies widely and may be related to the site of the mutation on the NGF receptor or whether there is genetic homo- or heterozygosity [55].

### Management

Medical management is supportive and oriented to control of hyperthermia, prevention of self-mutilation and treatment of orthopedic problems that potentially can cause severe and debilitating deformities. In addition it is also necessary to help families cope with behavior and educational issues. Febrile spikes will respond to use of acetaminophen and/or ibuprofen or direct cooling in a bath or cooling blanket. Diazepam, which is used for FD patients, has not been as effective in CIPA patients; chlorpromazine or chloral hydrate is more effective in relaxing the children and allowing them to cool. Careful daily inspection for unrecognized injury is important. Braces may be required on the ankles to prevent injury to these weight bearing joints. Some children will require smoothing of the teeth or extraction to prevent self-mutilation of the tongue and lips. Irritability, hyperactivity, and susceptibility to rages are seen in about 50% of patients. Chlorpromazine has been helpful as well as behavior modification. The prognosis for independent function depends on the degree of disease expression and the ability to control the secondary clinical problems.

## Prognosis and goals

There are a number of hereditary and sensory and autonomic neuropathies (HSAN). Identification and classification of these entities is an ongoing process and will be refined as we become more exact in clinical acumen, definitive autonomic testing, pathological examinations and finally molecular evaluations. As genetic tests for specific diagnosis are developed, genetic counseling will be more specific and we will gain insight as to how to provide more definitive therapies.

Prognosis for the HSAN disorders is improving and they can no longer be considered only as diseases of childhood. With greater understanding of the various disorders and development of treatment programs, increasing numbers of patients are reaching adulthood. The goal is now to help them achieve independent function with minimal physical limitations. As the disorders are so rare, centralization of care increases our ability to diagnose patients accurately, understand the natural progression of the disorders and to recommend appropriate interventions and treatments. We anticipate that further improvement in prognosis will be based on increased understanding of specific gene actions that will lead to definitive therapies.
